# Tumoral form of aspergillosis in central nervous system (cerebral aspergilloma): case report

**DOI:** 10.1590/S1516-31802003000600007

**Published:** 2003-11-06

**Authors:** Eberval Gadelha Figueiredo, Erich Fonoff, Marcos Gomes, Emílio Macedo, Raul Marino Júnior

**Keywords:** Aspergillosis, Diabetes mellitus, Aspergillus, Central nervous system neoplasms, Aspergilose, Diabetes mellitus, Aspergillus, Tumores cerebrais, Sistema nervoso central

## Abstract

Aspergillosis of the central nervous system is an uncommon infection, mainly occurring in immunocompromised patients. It may be presented in several forms: meningitis, mycotic aneurysms, infarcts and the tumoral form (aspergilloma). The authors report a case of a diabetic patient with cerebral aspergilloma.

## RESUMO

Aspergilose do sistema nervoso central é uma infecção rara, acometendo principalmente pacientes imunossuprimidos. Clinicamente pode se apresentar sob várias formas: meningite, aneurisma micótico, infartos cerebrais e como lesão tumoral (aspergiloma). Os autores apresentam o caso de um paciente diabético com aspergiloma cerebral.

## INTRODUCTION

Aspergillosis of the central nervous system is an uncommon infection, mainly occurring in immunocompromised patients. There are various clinical presentation forms of this infection: aseptic^1,2^ and persistent meningitis, mycotic aneurysms,^2^ ischemic and hemorrhagic infarcts and the tumor-like form or aspergilloma. In this article we present a case of a patient with cerebral aspergilloma.

## CASE REPORT

A forty-two-year-old woman was brought to the emergency department. She had diabetes that was under poor control and she had been having progressive headaches over a three-week period. Her mental state was impaired, and she had right-sided body weakness and loss of vision. The neurological examination showed right hemiparesis, decreased visual acuity in the right eye and blindness in the left eye. There was no eye deviation or anisocoria. Further examination revealed comprehension aphasia, thus accounting for the impairment of her mental state.

**Figure 1:**
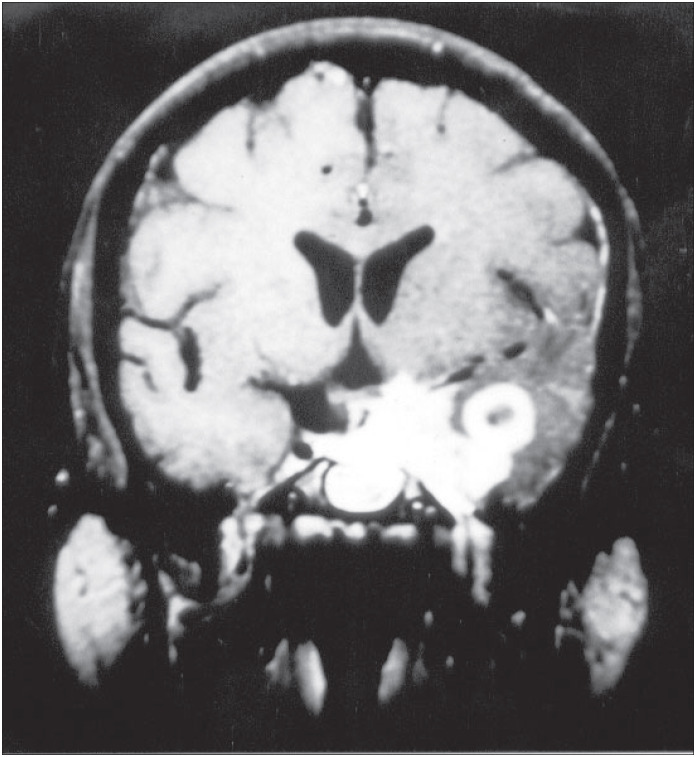
Coronal magnetic resonance imaging of the brain in the T1-weighted series after gadolinium injection, showing a lesion over the left temporal lobe in its mesial aspect and infiltrating the left cavernous sinus, with high signal.

**Figure 2:**
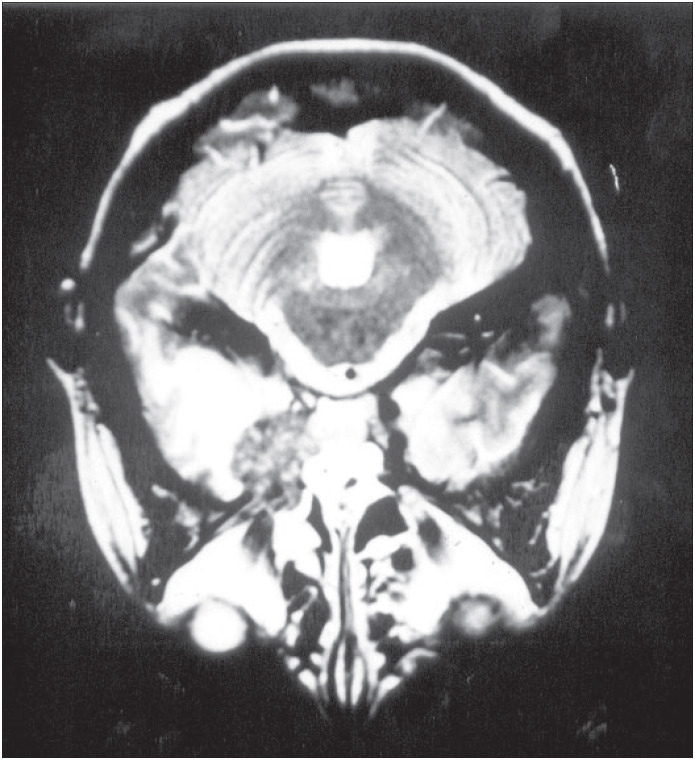
Magnetic resonance imaging of the brain in the T2 series, showing left temporal lobe edema.

A computed tomographic scan revealed a tumor-like lesion with high density and moderate mass effect on the anterior left temporal lobe with edema in the surrounding area. It appeared to have moderate heterogeneous enhancement with iodide contrast solution. Magnetic resonance imaging showed also a mass over the left temporal lobe in its mesial aspects and infiltrating the left cavernous sinus, with a high signal in the T1-weighted series (Figure 1). The surrounding edema was best observed in the T2 series (Figure 2).

After compensating for her diabetes, she underwent surgery by means of temporoparietal craniotomy. The lesion encountered had a light yellowish color, hard consistency and very significant bleeding when manipulated. The intraoperative pathological examination revealed fungal hyphae. The operation became protracted and resection was only partially performed.

During the postoperative period, she was given intravenous amphotericin B. She required long treatment due to postoperative fungal meningitis that was probably elicited by the surgical manipulation.

## DISCUSSION

Cerebral aspergillosis is an infrequent medical condition, but it is still a potentially fatal disease. However, it has become more frequently diagnosed because of high rates of suspicion among immunocompromised patients such as those with transplants and HIV-positive or aids patients, who show higher incidence of the condition. In a series of 1,730 transplanted patients, 60 were neurologically compromised and 18.3% of these had aspergilloma.^2^ A few more cases with the tumoral form in immunocompromised patients have been reported by Shuper.^3^

The lungs are probably the entry portal. However, isolated central nervous system aspergillosis (no detectable pulmonary disease) has already been reported.^4^ The prognosis in such patients is very poor even with surgical and anti-fungal treatment, especially when there is an association with other bacteria or infections.^1,4^

Aspergilloma is even rarer in patients who are apparently immunocompetent, although all cases described have had associated diseases such as diabetes mellitus or unhealthy habits like drug abuse.^1,4^

Diagnosis of aspergilloma in immuno-competent patients remains difficult because medical staff rarely suspects this condition. Although such patients have much better prognosis than for immunocompromised patients, the diagnosis is frequently missed or delayed.^3^

Neuroimaging gives some backing for considering such a diagnosis, although it does not bring any specific findings to light. The radiological findings in aspergilloma cases are the same as for brain abscesses and may have an appearance similar to that of many tumors. There is frequently a ring-like enhancement when contrast medium is injected. With magnetic resonance imaging, it appears as a tumoral lesion with a low signal in the T1-weighted series, with variable edema on the lesion periphery. Routine analysis of cerebrospinal fluid does not help in elucidating the etiology.
